# Pancreatic Extragastrointestinal Stromal Tumor: A Case Report

**DOI:** 10.7759/cureus.54514

**Published:** 2024-02-20

**Authors:** Tianyu Song, Qiang Hong, Yulian Wu

**Affiliations:** 1 Department of Surgery, Fourth Affiliated Hospital, Zhejiang University School of Medicine, Yiwu, CHN; 2 Department of Surgery, Second Affiliated Hospital, Zhejiang University School of Medicine, Hangzhou, CHN

**Keywords:** complementary therapy, spindle cell, pancreatic extragastrointestinal stromal tumor (egist), pancreas, gastrointestinal stromal tumor (gist)

## Abstract

Gastrointestinal stromal tumors (GISTs) are soft tissue sarcomas that originate from the mesenchymal cells of the gastrointestinal tract. Extra-GISTs (EGISTs) are caused by sites outside the gastrointestinal tract. We reported a case of EGIST of the pancreas in a 51-year-old woman. Enhanced CT scan showed a rounded, slightly hypointense focus in the head of the pancreas and the right pars compacta of the descending duodenum. Routine laboratory and endocrine tests were unremarkable. The patient underwent laparoscopic surgery. The diagnosis of EGIST was confirmed through histopathological and immunohistochemical examination. The tumor was found to be CD117+, CD34+, and DOG+ with a high risk of malignancy. No recurrence was observed during the nine-month postoperative follow-up.

## Introduction

Gastrointestinal stromal tumors (GISTs) are mesenchymal tumors that can develop anywhere in the gastrointestinal tract. They account for less than 1% of all gastrointestinal tumors [1-3]. They can occur throughout the gastrointestinal tract from the esophagus to the anus. GISTs usually affect older patients and are most frequently found in the stomach (60%-70%), small intestine (20%-25%), colon and rectum (5%), and esophagus (<5%) [4,5]. Gastrointestinal mesenchymal tumors are referred to as extragastrointestinal stromal tumors (EGISTs) when they arise outside of the gastrointestinal tract and account for approximately 5%-10% of all GISTs [6]. EGISTs arising from the pancreas are much rarer, with approximately 30 cases reported in the literature to date [7].

## Case presentation

**Figure 1:**
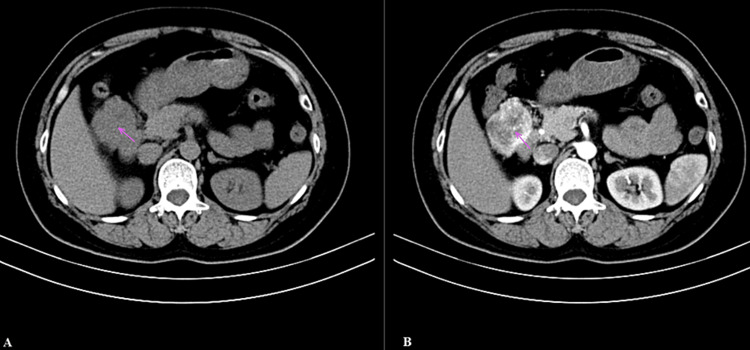
The imaging features of the mass on computerized tomography scan and enhanced scan. (A) A round, slightly less dense lesion. (B) Enhanced scan indicates inhomogeneous enhancement.

A 51-year-old woman presented with edematous erythema of the cheeks in March 2023. Following her hospitalization, she requested a full-body examination. An abdominal ultrasound unexpectedly revealed a hypoechoic mass over the right kidney. She did not report any discomfort such as abdominal pain, bloating, jaundice, nausea and vomiting, or wasting. Routine laboratory tests, serum tumor markers, and hormone levels were all within normal limits. Contrast-enhanced computed tomography (CT) of the abdomen revealed round, slightly hypodense foci in the head of the pancreas and the right parasternal aspect of the descending duodenum, measuring approximately 57 × 46 mm (Figure 1A). The enhancement scan showed inhomogeneous marked enhancement with poor local delineation from the pancreatic head and descending duodenum (Figure 1B).

**Figure 2:**
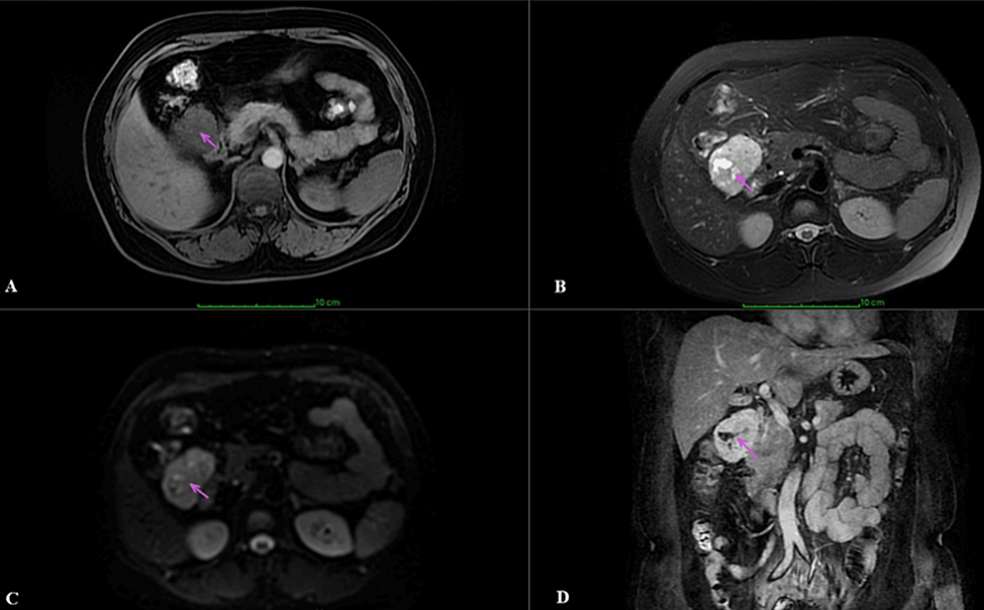
The imaging features of this mass on magnetic resonance imaging. (A) Low signal intensity lesion on T1-weighted image. (B) High signal intensity lesions on T2-weighted images. (C) High signal intensity lesion on DWI image. (D) Coronal T1-weighted enhancement scan with marked inhomogeneous enhancement.

Subsequently, she underwent a magnetic resonance (MR) enhanced scan of the abdominal mass. On MR imaging (MRI), the mass showed hypointensity on T1-weighted images (Figure 2A) and hyperintensity on T2-weighted images (Figure 2B), with patchy fluid signal shadows seen internally. The mass showed high intensity on diffusion-weighted imaging (DWI) images (Figure 2C), and the enhanced scan of the lesion after injection of gadolinium contrast agent showed marked inhomogeneous enhancement (Figure 2D).

**Figure 3:**
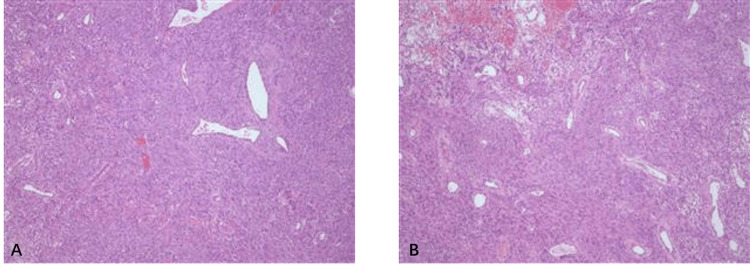
Microscopic findings of the mass. (A, B) The tumor is mainly composed of spindle cells and epithelioid cells.

In March 2023, the patient underwent laparoscopic surgery, during which an exophytic mass of approximately 6*5 cm was found in the head of the pancreas. The mass was meticulously separated from the surrounding tissue and found to occupy the entire head of the pancreas without invading the duodenum. And the tumor was removed intact. The postoperative course was uneventful. The histopathological examination revealed a mixed cell type tumor, primarily composed of spindle and epithelioid cells with focal atypia (Figures 3A, 3B). The mitotic count was 3/50 high-power fields (HPF). The immunohistochemical examination indicated that the tumor cells were positive for CD117, CD34, and DOG-1 antibodies.

Based on the site of growth of the mass and the margins of the surgical specimen, the final diagnosis of pancreatic GIST was made. According to the National Institutes of Health (NIH) 2008 Modified Chinese Consensus 2017 revision, pancreatic GIST was diagnosed as a high risk. The patient was started on imatinib mesylate after surgery, and no radiographic evidence of recurrence was observed during the nine-month follow-up period.

## Discussion

We report a rare case of primary pancreatic GIST. EGISTs are GISTs that are reported as primary tumors outside of the gastrointestinal tract [4,5]. The incidence of EGIST has been reported to be approximately 5%-10% of all GISTs [8]. The median age at diagnosis was 55 years, and the male-to-female ratio was 1:1.04 [9].

Symptoms of EGIST are mainly related to tumor growth and can lead to acute abdomen with bowel obstruction, abdominal distension or rupture, and bleeding [10,11]. Most small EGISTs, which are less than 5 cm in size, are asymptomatic and are typically discovered incidentally through imaging or palpation [9]. EGISTs should be considered potentially malignant due to the high risk of recurrence and metastasis [9]. EGIST diagnosis is based on cytomorphology, immunohistochemistry, and molecular genetic testing. Hematoxylin-eosin (HE) staining results differentiate three types of GIST: fusiform, epithelioid, or mixed. Fusiform is the most prevalent type [10]. On immunostaining, CD117 (>95%) and CD34 (70%) were strongly present and positive, while smooth muscle actin (30%), S-100 (5%), junctional proteins (2%), and cytokeratins (2%) were also reported [8,12-14]. Furthermore, DOG-1 is a protein expressed in nearly all GISTs and can serve as a biomarker for this type of tumor [10]. Molecular genetic testing, such as mutations in genes like CD117 (KIT, a type III receptor tyrosine kinase), can predict a tumor's sensitivity to molecularly targeted therapies. This testing may be used for initial screening.

For preoperative staging of EGIST, contrast-enhanced computed tomography of the abdomen and pelvis and contrast-enhanced computed tomography of the chest are the tests of choice [15]. Medical imaging data is crucial in determining the size and location of the tumor, identifying distant metastases, and detecting invasion of adjacent anatomic structures [16]. Surgery remains the most viable option for the treatment of EGIST, and the primary goal of EGIST treatment is complete resection of the tumor with clear margins. Lymph node dissection is necessary when preoperative imaging indicates lymph node infiltration. For patients with large tumors, open surgery is the preferred surgical approach due to the risk of tumor rupture and peritoneal spread associated with laparoscopic surgery [15].

About 20%-30% of GISTs are high-risk or malignant. Postoperative adjuvant therapy is indicated based on tumor size, mitotic count, location, and whether the tumor is ruptured or not [17,18]. In high-risk patients, in addition to surgery and targeted therapy, follow-up with serial CT scans approximately 10 years after imatinib initiation is recommended [19]. A younger age at diagnosis is typically associated with a worse prognosis and larger tumor size. Therefore, clinicians need to consider EGIST in the differential diagnosis of abdominal tumors outside the gastrointestinal tract to improve diagnosis rates and increase patients' chances of survival.

## Conclusions

GISTs are rare, with the majority originating in the stomach. In rare cases, they may be located in the pancreas, where they have a higher risk of recurrence and poorer prognosis. Surgical resection is required for pancreatic GIST, and postoperative adjuvant therapy is determined by the patient's level of risk. Lifelong follow-up is usually necessary for patients. Further research is needed for the diagnosis and treatment of pancreatic GIST.
